# The Impact of Peripheral Vision on Manual Reaction Time Using Fitlight Technology for Handball, Basketball and Volleyball Players

**DOI:** 10.3390/bioengineering10060697

**Published:** 2023-06-07

**Authors:** Dana Badau, Alina Mihaela Stoica, Marin Florin Litoi, Adela Badau, Daniel Duta, Cezar Gheorghe Hantau, Anca Maria Sabau, Bogdan Marian Oancea, Catalin Vasile Ciocan, Julien Leonard Fleancu, Bogdan Gozu

**Affiliations:** 1 Petru Maior Faculty of Sciences and Letters, George Emil Palade University of Medicine, Pharmacy, Sciences and Technology, 540142 Targu Mures, Romania; 2 Interdisciplinary Doctoral School, Transylvania University of Brasov, 500036 Brasov, Romania; 3 Department of Physical Education and Sports, University of Bucharest, 050107 Bucharest, Romania; 4 Faculty of Physical Education and Sport, Ovidius University of Constanta, 900470 Constanta, Romania; 5 Faculty of Physical Education and Sport, National University of Physical Education and Sport, 060057 Bucharest, Romania; 6 Faculty of Geography, Tourism and Sports, University of Oradea, 410081 Oradea, Romania; 7 Faculty of Physical Education and Mountain Sports, Transylvania University of Brasov, 500036 Brasov, Romania; 8 Faculty of Physical Education and Sports, Vasile Alecsandri University of Bacau, 600115 Bacau, Romania; 9 Faculty of Sciences, Physical Education and Informatics, University of Pitesti, 110040 Pitesti, Romania

**Keywords:** manual execution, visual stimuli, unilateral and bilateral peripheral vision, fitlight technology, sport technology, team sports, junior players, training program, male and female players

## Abstract

The purpose of the research was to identify the impact of peripheral (unilateral and bilateral) vision on manual reaction time to visual stimuli in handball, basketball and volleyball players by implementing a 6-week experimental program of specific exercises and some adapted tests using Fitlight technology. The research included 412 players (212 male—51.5%; 200 female—48.5%) from three team sports: basketball—146 (35.4%), handball—140 (40%) and volleyball—126 (30.6%). The experimental program carried out over 6 weeks was identical for all handball, basketball and volleyball players participating in the study; two training sessions per week were performed, with each session lasting 30 min; 15 exercises were used for the improvement of manual reaction time to visual stimuli involving peripheral vision. Through the Analysis of Variance (ANOVA), we identified statistically significant differences between the arithmetic means of the samples of handball, basketball and volleyball players, as well as according to general samples also of gender (male and female), *p* = 0.000. Male and female handball samples achieved the greatest progress in manual reaction time to visual stimuli involving peripheral vision for the Reaction time test with a unilateral right visual stimulus (30 s) and the Reaction time test with a unilateral left visual stimulus (30 s), while general sample also of male and female basketball samples, for the Reaction time test with bilateral visual stimuli (30 s) and the Reaction time test with six Fitlights (1 min); male and female volleyball samples recorded the lowest progress in all tests compared to handball and basketball groups. According to our results, female samples made greater progress in reaction time than male groups for all four tests of the present study. The implemented experimental program led to an improvement in manual reaction time to visual stimuli due to the use of Fitlight technology and the involvement of peripheral vision for all research samples.

## 1. Introduction

Playing team sports requires athletes to have a complex level of physical, technical and tactical training, and the degree of sports mastery is influenced by the experience gained and the development of specific physical and technical skills [1,2]. In sports, the degree of technicality is influenced by the development level of human analyzers: visual [3,4], auditory [5,6], kinesthetic [7,8], tactile [9,10], and by the interrelation between human perceptions in the performance of sports skills [11,12]. The speed with which technical skills specific to team sports are performed is essential for optimizing athletic potential and is conditioned by the interconnection between the development level of manual or foot reaction time in relation to visual and spatiotemporal perceptions [13,14].

Clarity of vision, visual acuity and the development level of peripheral vision can have a major impact on increasing the efficiency of technical performance specific to team sports. The conditioning factors of vision are as follows: visual field, eye movement, eye health and integrity, eye size and shape, visual acuity, color perception, pigmentation, and the amount of photoreceptor cells present in the eyeball (rods and cones) [15,16]. Visual acuity refers to the clarity of vision and depends on the sensitivity of the nervous system, the sharpness of an image on the retina and the ability of the central nervous system (CNS) to analyze and interpret the information received and transmitted by the optic nerve [17,18]. Studies have demonstrated that there are two types of vision: central and peripheral [19]. Central, focused vision is formed in the center of the eye, being responsible for the detailed detection of visual information located straight ahead, in front of the human body [20]. Peripheral vision makes it easier to see static or moving objects, scenes or details around (outside central vision), from one side to the other (left and right sides) without moving the head or eyes [21] through the cooperation of nerve cells with the rod-shaped photoreceptor cells located outside the macula [22,23]. The typology of peripheral vision includes three categories: far-peripheral vision—between 60 and 110 degrees of the visual field; mid-peripheral vision—between 30 and 60 degrees of the visual field; near-peripheral vision—between 18 and 30 degrees of the visual field [24,25]. Peripheral vision is not equal for both eyes; several studies identified differences between the right eye and the left eye due to the lateralization of cerebral hemispheres [26,27]. Studies have shown that the visual field of the human eye spans approximately 170 degrees, while peripheral vision is limited to 90–100 degrees of the total visual field [28,29].

In sports activity, the interconnection between peripheral vision and brain plasticity contributes to the identification and detailed perception of the shapes and movements of objects in space without the linear focus of vision [30,31] but also to the development of anticipation ability and human reaction speed [32,33,34,35]. Studies have highlighted that the speed with which the information collected by peripheral vision is transmitted to the CNS is 25% faster than the information provided by central vision [34,35]. Objects or scenes that are not mainly on the right focus line make the brain react to visual stimuli more quickly, and this action is achieved through the permanent, continuous use of peripheral vision [32,36]. In the case of visual stimuli, human motor reaction time is influenced by a number of factors, such as the intensity of the visual stimulus, reflex arc time, level of visual acuity, level of peripheral vision and eye health [22,37].

The optimization of motor potential for team sports athletes is influenced by the development level of peripheral vision, manual reaction time, as well as sports experience and skills. A number of studies have assessed peripheral vision or central (foveal) vision in athletes, highlighting that the higher the degree of peripheral vision, the faster the brain processes information, and objects become clearer and more visible than in the case of foveal, linear vision [22,37]. Studies in the field of sports have identified that vision plays a major role in human perceptual ability [38,39]. We believe that, in team sports, the clarity of vision correlated with the level of peripheral vision can influence both the efficiency of technical skills and the performance potential of each player.

Peripheral vision has been analyzed in numerous studies from the perspective of its impact on postural control [40], movement change detection [41,42], the development of proprioceptive components and reaction time [43,44], spatial orientation, etc. [45,46]. Few studies have focused on assessing the role of vision in achieving athletic performance in different individual sports [47,48] or team sports [49,50,51], but we have not found any study aimed at identifying the impact of peripheral vision on manual reaction time by comparing three team games. In team sports, peripheral speed and reaction time are major factors for sports performance optimization [2,48,49,50]. The innovative aspects of the present study reside in the analysis of differences between handball, basketball and volleyball players regarding the development level of manual reaction time, the design of an experimental program using Fitlight technology, the adaptation of exercises so that they involve the response of peripheral vision to visual stimuli and the design of four tests adapted to the main purpose of the study. We believe that our research will contribute to the expansion of knowledge about the ways of improving manual reaction time through exercises involving peripheral vision and Fitlight technology. In the present study, we opted for the selection of basketball, handball and volleyball games because the ball is handled directly (without the use of other equipment) and predominantly manually; the technical executions require a continuous adaptation of the execution parameters and implicitly of the peripheral vision and reaction time in relation to teammates, opponents, the trajectory and speed of the ball and the playing space, etc.

The main purpose of the research was to identify the impact of peripheral (unilateral and bilateral) vision on manual reaction time to visual stimuli in handball, basketball and volleyball players by implementing a 6-week experimental program of specific exercises and some adapted tests using Fitlight technology. The secondary purpose was to identify differences in progress between male and female handball, basketball and volleyball samples regarding the improvement of manual reaction time to visual stimuli involving peripheral vision as a result of implementing a 6-week experimental program of specific exercises and some adapted tests using Fitlight technology.

## 2. Materials and Methods

### 2.1. Participants

The present cross-sectional study included 412 active junior players (212 male—51.5%; 200 female—48.5%) from three team sports: handball, basketball and volleyball. A total of 421 players were initially recruited for this study, of which 412 (97.8%) players were included in the study, and 9 (2.2%) players (6 males and 3 females) were excluded from the study due to injuries and could not perform the final tests. Player distribution: for basketball, 146 (35.4%) players (76 male, 70 female); for handball, 140 (40%) players (72 male, 68 female); for volleyball, 126 (30.6%) players (64 male, 62 female). Characteristics of all experimental groups: average age ± SD 17.43 ± 2.35, average sports experience ± SD 8.32 ± 1.7; for the male group: average age ± SD 17.51 ± 1.42, average sports experience ± SD 8.17 ± 1.97, height 191.37 ± 5.23 cm, peripheral visual field 107.55 degrees (right eye) and 107.47 degrees (left eye); for the female group: average age ± SD 17.35 ± 1.85, average sports experience ± SD 8.47 ± 2.14, height 172.72 ± 4.72 cm, peripheral visual field 108.43 degrees (right eye) and 108.40 degrees (left eye). The players included in the study were registered with four sports training centers from the cities of Brasov, Targu-Mures, Bucharest and Constanta. The athletes involved in the study were selected from the clubs with which the authors of the study collaborate based on existing protocols at the institutional level, which allowed direct access to the training process. The subjects were interviewed through direct communication on a voluntary basis. Due to the characteristics of physical development as a result of the impact of practicing a team sports game, we selected adolescents aged 16–18 years for the present study. During the adolescent period of 16–18 years, the processes of cognitive analysis and synthesis become finer [52,53,54,55,56]. Studies have shown that faster physical development has major influences on social and cognitive development [53,54,55,56,57,58]. The cognitive gap between 16- and 18-year-old girls and boys with accelerated physical development of the same age is smaller, as is the case with team sports athletes (where height is an important selection criterion) [56,57,58]. The adolescent develops the necessary mental tools, such as follows: the structures of formal logical thinking are developed and strengthened; the ability to interpret, evaluate, plan, anticipate, and make predictions; the critical and self-critical spirit; the system character of thinking is developed. The period of adolescence is the period of maximum growth of perceptive and representational capacities. Sensory activity increases, and the minimum and maximum thresholds of various analyzers and differential thresholds change. Visual acuity, accommodation capacity and distance vision (degree of distance) increase, and the field of vision widens. In adolescence, the processes of perception are also restructured. The adolescent has a complex, voluntary and persistent perception [53,54,55,56,57,58].

Inclusion criteria: age between 16 and 18 years, active junior 1 athlete, playing one of the three team games selected for the study, minimum 6 years of competitive sports experience, sub-elite sports level annual medical clearance for playing a competitive sport, no eye problems and the peripheral visual field of at least 90 degrees. Exclusion criteria: failure to complete the experimental sports training program, becoming injured during the experiment and needing physical rest and failure to complete the 4 proposed tests for the assessment of reaction time to visual stimuli. Peripheral vision was measured for the right eye and the left eye using the Bernell Vision Disc [59].

### 2.2. Study Design

The study was conducted from September to December 2022 and included an initial test followed by the implementation of the experimental program aimed at improving reaction time with the involvement of peripheral vision and the use of Fitlight technology over 6 weeks, and ended with a final test immediately after completion of the experimental program. Considering that the athletes came from different sports training centers, the study took place during the aforementioned period for each sports team; after completing the recording of all final tests, the results were summarized. Each of the two test sessions (initial and final testing) applied in this study included 4 motor tests aimed at assessing the development level of manual reaction time to visual stimuli with the involvement of peripheral vision. The order and period of application of the tests were identical for all participating athletes: the Reaction time test with unilateral right visual stimulus, the Reaction time test with unilateral left visual stimulus, the Reaction time test with bilateral visual stimuli, and the Reaction time test with 6 Fitlights. The tests allowed two attempts for each athlete, and the best result was taken into account.

All authors contributed equally to the present study. Moreover, all players voluntarily participated in the study based on informed consent respecting the principles of the Helsinki Declaration. The study was approved on 27 July 2022 by the Review Board of the Physical Education and Sports Program of G.E. Palade University of Medicine, Pharmacy, Science, and Technology of Targu Mures.

### 2.3. Fitlight Technology Used in the Physical Training and Testing Process

In this study, Fitlight technology was used in both the physical training and testing process. The experimental program included specific exercises that were adapted so as to contribute to the development of peripheral vision and reaction time to visual stimuli. The testing process consisted of designing and applying 4 tests for the assessment of manual reaction time to visual stimuli with the involvement of peripheral vision. Fitlight technology was developed for the purpose of training and measuring various motor skills specific to different physical and sports activities [60].

This technology incorporates wireless LED spots that operate as a patented sensor designed to react to tactile or kinetic impact when performing movements. Fitlight technology is equipped with preset programs that can be selected by coaches and athletes but also allows for the creation of customized programs. The spots can be arranged in various ways, being easy to handle and use on different panels or devices; they have a range of 50–75 m calculated up to the wireless system. Fitlight technology is available in packages of 4 to 32 spots. For the present research, we used two packages of 8 spots each, including the preset software. Training and testing results were recorded by the Fitlight software, which provides reliable real-time information that can be viewed and downloaded using the default applications accessible on laptops. Fitlight technology facilitates real-time monitoring and optimization of physical parameters as a result of physical programs or motor tests that use visual stimuli. The experts who designed Fitlight technology aimed to develop the following physical fitness components: reaction time, general coordination, hand/foot–eye coordination, execution and reaction speed, peripheral vision, spatial orientation, fine motor control, cognitive reaction time, etc. [61,62,63].

### 2.4. Experimental Study Program

**Figure 1:**
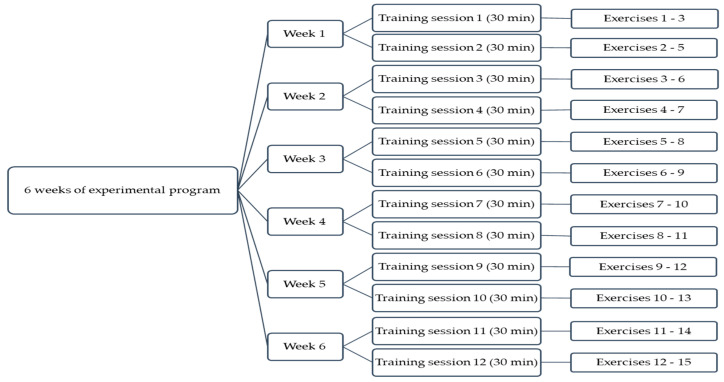
Experimental program planning.

The experimental program took place over a 6-week period for each of the handball, basketball and volleyball samples participating in the study; two training sessions per week were performed, with each session lasting 30 min. The experimental program included 15 exercises for the improvement of manual reaction time to visual stimuli involving peripheral vision and the use of Fitlight technology. The program was planned and structured as shown in Figure 1.

The exercises included in the experimental program were designed by taking into account the age characteristics and the level of sports training and were identical for all experimental samples, regardless of gender or the sport played. In addition, the exercises were designed to allow using Fitlight technology for the development of manual reaction time with the involvement of peripheral vision; the selected exercises were specific and common to the three team games (handball, basketball, volleyball) where the ball is predominantly manipulated by hand (except for volleyball, where it can also be hit with other parts of the body.

### 2.5. Measures

Each of the two test sessions (initial and final testing) applied in this study included 4 motor tests aimed at assessing the development level of manual reaction time to visual stimuli with the involvement of peripheral vision and the use of Fitlight technology. 

Tests 1/2—The Reaction time test with the unilateral right (Test 1)/left (Test 2) visual stimulus (30 s): On a tripod with a height of 1.4 m for female groups and 1.8 m for male groups, one Fitlight (stimulus spot) is placed, and 4 other spots are arranged in the corners of a vertical rectangular panel of 80/40 cm mounted at a maximum height of 1.6 m for female groups and 1.8 m for male groups (according to the average height of the two samples by gender). The tripod with the stimulus spot is placed on the right/left side of the player at a distance of 1 m and at a 90-degree angle relative to the participant’s directional orientation and gaze. The test lasts 30 s and consists in touching as many spots as possible on the vertical panel, whose color is identical to that of the stimulus spot. The spots may have different colors, but for this test, the number of touched spots of the same color as the stimulus spot is quantified. The starting position is standing upright with the face and both eyes towards the panel and the hands flexed with the palmar surface oriented towards the center of the panel, 50 cm away from it. During the test, the spot that lights up can be touched with any hand, but the head and eyes are not allowed to turn to the side.

Test 3—The Reaction time test with bilateral visual stimuli (30 s): On two tripods with a height of 1.4 m for female groups and 1.8 m for male groups, one Fitlight (stimulus spot) per device is placed, and 4 other spots are arranged in the corners of a vertical rectangular panel of 80/40 cm mounted at a maximum height of 1.6 m for female groups and 1.8 m for male groups (according to the average height of the two samples by gender). The tripods with the stimulus spots are placed bilaterally to the player at a distance of 1 m and at 90-degree angles to the right and left sides relative to the participant’s directional orientation and gaze. The test lasts 30 s and consists in touching as many spots as possible on the vertical panel, whose color is identical to that of the stimulus spots. The spots may have different colors, but for this test, the number of touched spots of the same color as the stimulus spots that will light up alternately and randomly is quantified. The starting position is standing upright with the face towards the panel and the hands flexed with the palmar surface oriented towards the center of the panel, 50 cm away from it. During the test, the spot that lights up can be touched with any hand, but the position of the head is not allowed to change.

Test 4—The Reaction time test with 6 Fitlights (1 min): The 6 Fitlights are placed on a vertical rectangular panel of 80/40 cm mounted at a maximum height of 1.6 m for female groups and 1.8 m for male groups (according to the average height of the two samples by gender). The 6 Fitlights are arranged three in a row on the small sides of the rectangle as follows: one up in the corner, another down in the corner, and the third in the middle of the distance between the two spots placed in the corners. During the test, the spots can be touched with either hand. The starting position is standing upright in front of the panel at a distance of 40 cm, keeping the hands flexed with the palmar surface oriented towards the center of the panel where a dot is drawn; the position of the head is straight, and the gaze is directed forward. Only the spot that lights up randomly must be touched, after which the participant returns to the initial position with the palms facing the center; the number of spots touched during 1 min is quantified.

### 2.6. Statistical Analysis

The results of the study were statistically processed using IBM-SPSS 22 software. In order to highlight the relevance of the obtained results, the following statistical parameters were calculated: arithmetic mean (X_It/Ft_); standard deviation (SD); mean difference between tests (∆X_Ft-It_); Student’s *t*-test (t), confidence interval with lower and upper bounds (95% CI); effect size (d). Interpretation of Cohen’s d effect size: 0.1–0.2, small; 0.3–0.5, medium; 0.5–0.8, large; over 0.8, very large. The mean differences between the three groups of male/female handball, basketball and volleyball players were calculated using the Analysis of Variance (ANOVA) for the following statistical parameters: SS—sum of squares, df—degrees of freedom; MS—mean square; F—F ratio; *p*—statistical probability value. For this study, the selected reference value for statistical significance was *p* < 0.05. Analysis of variance (ANOVA) was used in the present study, recommended by statisticians, for the three data samples in order to provide information about the relationship between the dependent variables in our case concretized in the experimental training program and the independent ones regarding the applied tests for the reaction time in relation to the peripheral vision.

## 3. Results

Table 1, Table 2, Table 3 and Table 4 show the most significant results for the four peripheral vision tests aimed at identifying the reaction time of active junior 1 handball, basketball and volleyball players. The results are presented in relation to the team sport played and are differentiated by gender.

Analysis of the Reaction time test with unilateral right visual stimulus highlights statistically significant differences (*p* = 0.001) between the final and initial test results for all samples of players differentiated by the general number and of gender and according to the sport played: handball, basketball, volleyball. All arithmetic mean differences for all tests and groups fell within the upper and lower bounds of the 95% CI. The arithmetic mean values for the two tests and the mean differences between the two tests applied in this study (Table 1) reveal that the progress made by female samples is greater than that of male samples for all three team sports. When analyzing the results in Table 1, it was found that, for male samples, the greatest progress is recorded by handball players with 4.394 executions, followed by basketball players with 4.373 executions, while the lowest progress is recorded by volleyball players with 3.714 executions. In the case of female samples, it was found that the results of the two test sessions for the Reaction time test with unilateral right visual stimulus are significantly better in statistical terms, with the greatest progress achieved by handball players with 4.604 executions, followed by basketball players with 4.450 executions, meaning 0.154 less than the handball sample; the lowest progress is recorded by volleyball players with 3.784 executions, so 0.82 less than the handball sample. The effect size values are greater than 0.8 for female handball and basketball groups, which reflects a very large effect, and for the volleyball sample, the effect size is large, ranging between 0.5 and 0.8. The effect size values for male handball, basketball and volleyball samples range between 0.5 and 0.8, which reflects a large effect; the highest score is achieved by male basketball players with 0.770, followed by handball players with 0.758, and the lowest effect size is for volleyball players with 0.551. The size effect for the general samples highlights a very large effect for handball and basketball and a large one for volleyball. 

Table 2 shows the results of the Reaction time test with unilateral left visual stimulus, where it can be found that the progress made by female groups is greater than that of male groups for all three team sports. The results obtained in this test are statistically significant (*p* = 0.001) for all samples of players according to the sport played: handball, basketball and volleyball. When analyzing the progress according to the team sport played, it was noted that the greatest progress is achieved by the general and male and female handball samples, followed by basketball players, while the lowest progress is recorded by male and female volleyball players. All arithmetic mean differences for all tests and groups fell within the upper and lower bounds of the 95% CI. The effect size values indicate that male and female handball and basketball samples, as well as the female volleyball sample, range between 0.5 and 0.8, which reflects a large effect; for male volleyball samples, the mean effect size value was medium 0.412; for general samples of all team sports, the effect size was large. 

The Reaction time test with bilateral visual stimuli required a more complex focus of peripheral vision because the two Fitlight visual stimuli were placed bilaterally. The results of all samples organized according to the team sport played emphasize a large effect size with values ranging between 0.509 and 0.752. The results obtained in this test reveal statistically significant progress (*p* = 0.001) for the general sample and also male and female handball, basketball and volleyball samples. When analyzing the progress made between the final and initial tests (Table 3), it could be observed that the greatest progress is achieved by the basketball samples, followed by handball samples; the lowest progress is recorded by the volleyball samples. It can also be noted that the progress made by female samples is greater than that of male samples. 

Regarding the Reaction time test with six Fitlights (1 min), the differences between tests were statistically significant (*p* = 0.001) for all samples of players: handball, basketball, volleyball. All arithmetic mean differences for all tests and groups fell within the upper and lower bounds of the 95% CI. When analyzing the results shown in Table 4, it was found that the progress made by female samples is greater than that of male samples for all three team sports. An analysis of the results obtained by male samples revealed that the greatest progress is achieved by basketball players with 6.114 executions, followed by handball players with 5.023 executions, while the lowest progress is recorded by volleyball players with 4.421 executions. In the case of female samples, it was noted that the greatest progress was achieved by basketball players with 6.317 executions, followed by handball players with 5.696 executions; the lowest progress was recorded by volleyball players with 4.750 executions. The effect size values are greater than 0.8 for female handball groups and male and female basketball groups, which reflects a very large effect, while for the other samples, the effect size is large, ranging between 0.5 and 0.8. The size effect for the general samples highlights a very large effect for handball and basketball and a large one for volleyball.

Through the Analysis of Variance (ANOVA), we aimed to identify the arithmetic mean differences between the samples of handball, basketball and volleyball players, as well as according to the general number of subjects and gender (male and female). The results shown in Table 5 reflect statistically significant differences between male and female handball, basketball and volleyball players for all four tests of the present study, with significance threshold values lower than *p* < 0.005, more specifically, ranging between 0.001 and 0.032. 

## 4. Discussion

The present study mainly focused on the improvement of manual reaction time to visual stimuli with the involvement of peripheral vision by implementing an exercise program based on the use of Fitlight technology for active junior 1 handball, basketball and volleyball players. The study also aimed to identify the differences between male and female samples in terms of reaction time to visual stimuli with the involvement of peripheral vision by implementing a 6-week program of adapted exercises using Fitlight technology.

A comparative analysis of the progress achieved according to the team sport played showed that general samples and also male and female handball samples made the greatest progress in manual reaction time to visual stimuli involving peripheral vision for the Reaction time test with a unilateral right visual stimulus (30 s) and the Reaction time test with a unilateral left visual stimulus (30 s). Additionally, general samples and male and female basketball samples had bilateral visual stimuli (30 s) for the Reaction time test and the Reaction time test with six Fitlights (1 min). In this study, volleyball samples recorded the lowest progress in all tests compared to handball and basketball groups. We believe that this is due to the different specifics of the technique and the way of organizing and playing the three team sports, which require different adaptations and manifestations of technical skills. Handball, basketball and volleyball are differently organized, so peripheral vision and reaction time are also differently adapted when performing technical skills during training and competitive games. 

The results of our study contribute to the expansion of knowledge about the ways of improving manual reaction time through exercises involving peripheral vision and Fitlight technology. Moreover, the results of this study are consistent with previous studies that have identified significant differences in reaction time between team games due to their structure and technical specificity [64,65].

According to our results, female samples made greater progress in reaction time than male groups for all four tests of the present study. The greater progress in reaction time for female groups compared to male groups may derive from gender characteristics, the motor and cognitive characteristics of the age between 16 and 18 years, and the increased focus of attention during the training and testing performed in this study. Through the obtained results, we can contribute to confirming the findings of previous studies that have also identified gender differences between team sports in correlation with the development level of various motor skill parameters [65,66]. 

Previous studies pointed out the impact of using Fitlight technology in different team sports to improve reaction time [67]. Numerous authors have shown that reaction time, peripheral vision, and manual and hand-eye coordination can be enhanced by implementing specialized training that uses information technologies [68]. There are studies that focused on examining the association between peripheral vision and reaction time in different categories of the population [69,70]. 

By the relevance of the results, our study is in line with previous research in the field of sports. Thus, a study conducted with 17 female basketball players analyzed their reaction time, anticipation time and peripheral vision using information technology and concluded that peripheral vision had an influence on both reaction time and anticipation time [71]. The effect of peripheral vision on reaction time following the implementation of software and information technologies was examined in a study involving 24 hockey players, and the results highlighted that their use significantly improved the levels of peripheral vision and reaction time [72]. Another study that included 360 active athletes of both genders revealed differences in the development of three types of reaction time for male and female basketball, handball and volleyball players, emphasizing that Fitlight technology could contribute to the improvement of manual reaction time to visual stimuli, the observed differences being due to the specifics of the sport played [65]. An interesting study on how visual stimuli could influence reaction time involved 38 football players who were divided into two groups that followed a 6-month exercise program using Fitlight technology; the authors concluded that the implemented program contributed to the improvement of reaction time [61]. Studies demonstrated that the relationship between peripheral vision and the optimization of human reactions is determined by brain plasticity, the distance and positioning of the stimulus, the type of sport and the sports experience [73,74]. A series of studies aimed at evaluating agility or reactive speed in handball, volleyball and basketball players or other team games highlighted superior results in basketball and handball players compared to other sports categories [2,75,76]. All the studies mentioned above highlight the relationship between the optimization of reaction time and the level of manifestation of human perceptions through the implementation of specialized training adapted to the purpose and specifics of the sport played. 

The strengths of the study include the relatively large number of athletes (general; male and female) involved in the study; the comparative analysis of three types of team sports (handball, basketball, and volleyball); the design and implementation of the experimental program; the design and application of the four tests for the assessment of manual reaction time to visual stimuli; the use of Fitlight technology in the sports training and testing process. 

The limitations of the study include the duration of only 6 weeks of the experimental program; failure to apply standardized tests; the involvement in the study of athletes aged only between 16 and 18 years; the evaluation focused on the number of manual reactions to visual stimuli over a predetermined period of time (30 s, 1 min.) and the speed of manual reaction to visual stimuli was not measured; and the lack of a control group, because we only aimed to compare the players of the three team games who are selected on specific physical and sporting criteria and are trained specifically for these sports and we consider that their comparison with a group of subjects from other sports (with other characteristics and sports selection criteria) or with a group of non-athletes will not provide relevant information for improving the specific performances of team sports games.

## 5. Conclusions

The experimental program implemented in the study demonstrated its effectiveness by determining the optimization of manual reaction time to visual stimuli through the use of Fitlight technology and the involvement of peripheral vision in performing specific exercises. A comparative analysis of the progress achieved according to the team sport played highlighted that handball and basketball samples made the greatest progress in manual reaction time to visual stimuli involving peripheral vision compared with volleyball samples, which recorded the lowest progress in all tests. According to our results, female samples achieved greater progress in reaction time than male groups for all four tests of the present study. Gender differences identified in the study are determined by the differences and peculiarities of cognitive and motor maturation. The use of Fitlight technologies in the training process in sports games can facilitate the optimization of technical and physical training and increase the attractiveness of the training. Fitlight technologies can be used in numerous sports and physical activities as equipment for practice and assessment; another major argument for the use of these technologies consists in providing information in real time.

Future research directions regarding the correlation between peripheral vision and human reaction time should focus on identifying gender differences for various age groups, differences between diverse 3D positions of the stimulus, the connection between fitness components and peripheral vision, the role of peripheral vision and reaction time in acquiring technical skills, etc.

## Figures and Tables

**Table 1:** Statistical analysis of the results obtained by handball, basketball and volleyball players for the Reaction time test with unilateral right visual stimulus.

Test	Sports	Gender (no.)	X_It_ ± SD	X_Ft_ ± SD	∆X_Ft-It_	95% CI Upper; Lower	*t*	*p*	d
Reaction time test with unilateral right visual stimulus (30 s)	Handball	M (76)	17.971 ± 5.104	22.366 ± 6.409	4.394	4.672; 4.115	31.462	0.001	0.758
		F (70)	18.024 ± 6.412	22.628 ± 4.684	4.604	4.975; 4.431	32.281	0.001	0.819
		General (146)	17.996 ± 6.147	22.491 ± 4.631	4.495	4.981; 4.037	31.739	0.001	0.825
	Basketball	M (72)	16.716 ± 6.050	21.089 ± 5.241	4.373	4.693; 4.053	27.276	0.001	0.770
		F (68)	16.892 ± 5.753	21.342 ± 6.732	4.450	4.821; 4.197	26.953	0.001	0.844
		General (140)	16.801 ± 5.849	21.211 ± 5.063	4.410	4.931; 3.873	25.956	0.001	0.806
	Volleyball	M (64)	16.555 ± 6.168	20.269 ± 7.244	3.714	4.041; 3.386	22.670	0.001	0.551
		F (62)	16.703 ± 6.325	20.487 ± 5.916	3.784	4.268; 3.516	24.418	0.001	0.617
		General (126)	16.627 ± 6.201	20.376 ± 6.719	3.749	4.184; 3.158	23.018	0.001	0.579

_It_—initial test; _Ft_—final test; X—arithmetic mean; SD—standard deviation; *t*—Student’s *t*-test, ∆X_Ft-It_—mean difference between final and initial testing; 95% CI—confidence interval with lower and upper bounds; d—effect size.

**Table 2:** Statistical analysis of the results obtained by handball, basketball and volleyball players for the Reaction time test with unilateral left visual stimulus.

Test	Sports	Gender (no.)	X_It_ ± SD	X_Ft_ ± SD	∆X_Ft-It_	95% CI Upper; Lower	*t*	*p*	d
Reaction time test with unilateral left visual stimulus (30 s)	Handball	M (76)	16.887 ± 4.161	20.436 ± 6.436	3.549	3.850; 3.247	23.479	0.001	0.654
		F (70)	16.947 ± 4.161	20.816 ± 5.723	4.069	4.327; 3.611	24.836	0.001	0.773
		General (146)	16.915 ± 4.581	20.618 ± 6.142	3.703	4.011; 3.461	23.215	0.001	0.683
	Basketball	M (72)	16.268 ± 6.950	19.776 ± 5.551	3.507	3.890; 3.124	18.283	0.001	0.557
		F (68)	16.441 ± 6.372	20.297 ± 6.814	3.856	4.289; 3.318	19.913	0.001	0.584
		General (140)	16.352 ± 5.623	20.029 ± 6.368	3.677	4.461; 3,515	19,284	0.001	0.612
	Volleyball	M (64)	16.301 ± 7.005	19.238 ± 7.226	2.936	3.300; 2.572	16.112	0.001	0.412
		F (62)	16.426 ± 6.471	19.629 ± 5.512	3.203	3.464; 2.781	17.582	0.001	0.532
		General (126)	16.362 ± 6.382	19.430 ± 5.435	3.068	3.748; 2.589	17,385	0.001	0.517

_It_—initial test; _Ft_—final test; X—arithmetic mean; SD—standard deviation; *t*—Student’s *t*-test, ∆X_Ft-It_—mean difference between final and initial testing; 95% CI—confidence interval with lower and upper bounds; d—effect size.

**Table 3:** Statistical analysis of the results obtained by handball, basketball and volleyball players for the Reaction time test with bilateral visual stimuli.

Test	Sports	Gender (no.)	X_It_ ± SD	X_Ft_ ± SD	∆X_Ft-It_	95% CI Upper; Lower	*t*	*p*	d
Reaction time test with bilateral visual stimuli (30 s)	Handball	M (76)	17.281 ± 6.145	20.887 ± 4.577	3.605	3.931; 3.279	22.045	0.001	0.665
		F (70)	18.014 ± 6.324	21.989 ± 5.376	3.975	4.412; 3.546	24.154	0.001	0.677
		General (146)	17.632 ± 5.369	21.415 ± 5.582	3.783	4.327; 3.267	22.894	0.001	0.690
	Basketball	M (72)	16.373 ± 5.058	20.300 ± 5.634	3.827	4.001; 3.252	19.330	0.001	0.714
		F (68)	17.065 ± 5.822	21.121 ± 4.921	4.056	4.662; 3.417	18.382	0.001	0.752
		General (140)	16.709 ± 5.146	20.698 ± 5.572	3.989	4.759; 3.214	18,582	0.001	0.743
	Volleyball	M (64)	15.952 ± 6.946	19.079 ± 5.201	3.126	3.504; 2.749	16.577	0.001	0.509
		F (62)	16.287 ± 6.428	19.539 ± 4.921	3.252	3.793; 2.817	17.921	0.001	0.568
		General (126)	16.116 ± 6.318	19.305 ± 5.047	3.189	3.647; 2.575	17.271	0.001	0.557

_It_—initial test; _Ft_—final test; X—arithmetic mean; SD—standard deviation; *t*—Student’s *t*-test, ∆X_Ft-It_—mean difference between final and initial testing; 95% CI—confidence interval with lower and upper bounds; d—effect size.

**Table 4:** Statistical analysis of the results obtained by handball, basketball and volleyball players for the Reaction time test with 6 Fitlights (1 min).

Test	Sports	Gender (no.)	X_It_ ± SD	X_Ft_ ± SD	∆X_Ft-It_	95% CI Upper; Lower	*t*	*p*	d
Reaction time test with 6 Fitlights (1 min)	Handball	M (76)	33.891 ± 7.276	38.914 ± 5.813	5.023	5.683; 4.612	44.314	0.001	0.762
		F (70)	34.571 ± 6.102	40.267 ± 6.879	5.696	6.121; 5.16	42.157	0.001	0.876
		General (146)	34.217 ± 5.877	39.562 ± 6.325	5.345	6.245; 5.045	41.744	0.001	0.875
	Basketball	M (72)	34.142 ± 8.714	40.256 ± 6.242	6.114	6.581; 5.478	42.285	0.001	0.806
		F (68)	35.892 ± 8.143	42.214 ± 6.598	6.317	6.614; 5.772	47.155	0.001	0.853
		General (140)	34.992 ± 8.049	41.207 ± 6.355	6.215	6.589; 5.538	45.215	0.001	0.857
	Volleyball	M (64)	33.173 ± 8.085	37.594 ± 6.815	4.421	4.916; 4.027	37.276	0.001	0.591
		F (62)	33.835 ± 8.253	38.585 ± 6.576	4.750	5.163; 4.217	40.263	0.001	0.636
		General (126)	33.498 ± 6.116	38.081 ± 5.325	4.583	5.205; 4.109	38.138	0.001	0.538

_It_—initial test; _Ft_—final test; X—arithmetic mean; SD—standard deviation; *t*—Student’s *t*-test, ∆X_Ft-It_—mean difference between final and initial testing; 95% CI—confidence interval with lower and upper bounds; d—effect size.

**Table 5:** ANOVA (Analysis of Variance) between handball, basketball and volleyball players.

Tests	Gender	Test Session	SS	df	MS	F	*p*
Reaction time test with unilateral right visual stimulus (30 s)	M	It	68.678	2	34.339	7.529	0.001
		Ft	156.460	2	78.230	15.097	0.001
	F	It	39.542	2	31.347	8.271	0.017
		Ft	42.145	2	21.167	5.981	0.032
	General	It	139.348	2	69.674	15.699	0.001
		Ft	289.673	2	144.836	27.997	0.001
Reaction time test with unilateral left visual stimulus (30 s)	M	It	37.997	2	28.999	6.170	0.017
		Ft	40.842	2	20.421	3.515	0.032
	F	It	16.553	2	7.147	5.276	0.021
		Ft	34.812	2	19.363	3.512	0.008
	General	It	36.645	2	18.323	4.424	0.013
		Ft	95.418	2	47.709	8.204	0.001
Reaction time test with bilateral visual stimuli (30 s)	M	It	45.651	2	22.825	3.891	0.022
		Ft	68.678	2	34.339	7.529	0.001
	F	It	64.784	2	32.392	7.818	0.001
		Ft	110.767	2	55.384	9.134	0.001
	General	It	129.849	2	64.925	15.797	0.001
		Ft	218.390	2	109.195	18.132	0.001
Reaction time test with 6 Fitlights (1 min)	M	It	124.713	2	41.315	9.362	0.001
		Ft	167.426	2	68.251	8.1454	0.001
	F	It	131.518	2	44.368	7.745	0.004
		Ft	129.526	2	52.386	9.694	0.001
	General	It	214.376	2	98.573	15.214	0.001
		Ft	291.622	2	126.362	17.829	0.001

M—male; F—female; SS—sum of squares; df—degrees of freedom; MS—mean square; F—F ratio; *p*—statistical probability value.
